# A body shape index and body roundness index: two new body indices to identify diabetes mellitus among rural populations in northeast China

**DOI:** 10.1186/s12889-015-2150-2

**Published:** 2015-08-19

**Authors:** Ye Chang, Xiaofan Guo, Yintao Chen, Liang Guo, Zhao Li, Shasha Yu, Hongmei Yang, Yingxian Sun

**Affiliations:** Department of Cardiology, the First Hospital of China Medical University, 155 Nanjing North Street, Heping District, Shenyang, 110001 People’s Republic of China

## Abstract

**Background:**

The Body Mass Index (BMI) has long been used as an anthropometric measurement. Waist circumference (WC) and waist-to-height ratio (WHtR) have been proposed as alternatives to BMI. Recently, two new anthropometric indices, the A Body Shape Index (ABSI) and Body Roundness Index (BRI) have been developed as possible improved alternatives to BMI and WC. The main research aim is to assess the capacity of the ABSI and BRI to identify subjects with diabetes mellitus (DM) and the secondary aim is to determine whether ABSI and/or BRI is superior to the traditional body indices (BMI, WC, and WHtR).

**Methods and Results:**

This cross-sectional study was conducted in the rural areas of northeast China from January 2012 to August 2013, and the final analysis included data obtained form 5253 men and 6092 women. 1182 participants (10.4 %) suffered from DM. Spearman rank test showed that BRI and WHtR showed the highest Spearman correlation coefficient for DM whereas ABSI showed the lowest. The prevalence of DM increased across quartiles for ABSI, BMI, BRI, WC and WHtR. A multivariate logistic regression analysis of the presence of DM for the highest quartile vs. the lowest quartile of each anthropometric measure, showed that the WHtR was the best predictor of DM (OR: 2.40, 95 % CI: 1.42–3.39 in men; OR: 2.67, 95 % CI: 1.60–3.74 in women, both *P* < 0.001), and the ABSI was the poorest predictor of DM (OR: 1.51, 95 % CI: 1.05–1.97 in men; OR: 1.55, 95 % CI: 1.07–2.04 in women, both *P* < 0.05). ABSI showed the lowest AUCs (AUC: 0.61, 95 % CI: 0.58–0.63 for men; AUC: 0.61, 95 % CI: 0.59–0.63 for women) for DM in both sexes, while BRI (AUC: 0.66, 95 % CI: 0.63–0.68 for men; AUC: 0.67, 95 % CI: 0.65–0.69 for women) had high AUCs for DM that equaled those of WHtR.

**Conclusions:**

Our results showed neither ABSI nor BRI were superior to BMI, WC, or WHtR for predicting the presence of DM. ABSI showed the weakest predictive ability, while BRI showed potential for use as an alternative obesity measure in assessment of DM.

## Background

Diabetes mellitus (DM) has become a global health problem, and its rates of morbidity and mortality have continued to gradually increase [1]. Currently, DM is not curable, and DM prevention and management has been the focus of attention. Therefore, developing means to efficiently identify populations at high risk for DM is an important first step towards adopting preventive measures. Several studies have found a strong and direct association between obesity and DM [2, 3], and the World Health Organization (WHO) has defined obesity according to anthropometric index as a body mass index (BMI) ≥ 30 [4]. For the simpleness and usefulness, anthropometric indices have been recommended for predicting DM in many clinical practices [5, 6]. However, the BMI has been criticized because it does not discern between fat mass and muscle mass, or reflect an individual’s fat distribution [7, 8]. Therefore, waist circumference (WC) and waist-to-height ratio (WHtR) have been suggested as alternative obesity indices which can modulate the limitations of BMI, and numerous previous studies have confirmed that they are superior to BMI as indicator of risks for mortality, cardiometabolic risk factors and cardiovascular diseases, including DM [9–17].

Recently, two new body indices have been developed [18, 19]. In 2012, Krakauer and Krakauer [18] developed a new index known as the A Body Shape Index (ABSI), which is calculated using the following equation: $\mathrm{ABSI} = \frac{\mathrm{WC}}{{\mathrm{BMI}}^{2/3}{\mathrm{Height}}^{1/2}}$ Krakauer and Krakauer found a positive correlation between ABSI values and abdominal deposition of adipose tissue. Furthermore, ABSI values appears superior to measurements of WC and BMI for predicting premature death. Other studies have also found that ABSI can predict morbidity and mortality during patient follow-up periods [20, 21]. However, it has been suggested that ABSI is a weaker predictor of cardiovascular disease (CVD) when compared to BMI [22]. In 2013, Thomas et al. [19] developed another new index termed the Body Roundness Index (BRI), which is calculated using the following equation: $\mathrm{B}\mathrm{R}\mathrm{I} = 364.2-365.5\times \kern0.5em \sqrt{1-\left(\frac{{\left(\mathrm{W}\mathrm{C}/\left(2\uppi \right)\right)}^2}{{\left(0.5\mathrm{height}\right)}^2}\right)}$ BRI values range from 1 to 16, and rounder individuals tend to have larger values. The mean BRI values for men and women were found to be 4.64 ± 1.88 and 5.16 ± 2.24, respectively. Up to now, only a few studies have been conducted using the BRI. Maessen MF et al. [23] found the BRI was capable of identifying both CVD and CVD risk factors, but was not superior to BMI or WC in this regard. However, we don’t know whether these two new anthropometric measures could identify subjects with the presence of DM in the rural population.

We conducted this population-based, cross-sectional study to assess the capacity of these two new anthropometric measurements (ABSI and BRI) to identify individuals with DM in the rural populations of northeast China. We also compared the attributes of five anthropometric measurements (ABSI, BMI, BRI, WC, and WHtR), and attempted to determine whether the ABSI and BRI were superior to measurements of BMI, WC, and WHtR.

## Methods

### Study population

This study was conducted in Liaoning Province, located in Northeast China. From January 2012 to August 2013, a representative sample of individuals aged ≥ 35 years was selected to participate in assessing two new body indices (ABSI and BRI) for purposes of identifying cases of DM in rural northeast China. The study employed a multi-stage, stratified random cluster-sampling scheme. In the first stage, 3 counties (Dawa, Zhangwu, and Liaoyang County) were selected randomly from the rural areas of Liaoning province, and in the second stage, one town was randomly selected from each of the 3 counties. In the third stage, 26 rural villages in 3 towns were randomly selected for inclusion in the study. All eligible permanent residents aged ≥ 35 years (a total of 14 016 individuals) in each village were invited to participate in the study. Of those, 11 956 participants agreed to participate and completed the present study. The study protocol was approved by the Ethics Committee of China Medical University (Shenyang, China), and all procedures were performed in accordance with good ethical standards.

### Data collection

A written consent was obtained in all participants after they had been informed of the study’s objectives, benefits, and medical procedures, and had signed a confidentiality agreement regarding personal information. Participants who were illiterate completed their Informed Consent with the aid of their proxy. Only participants with a complete set of data for the variables analyzed in the present study were included in the final analysis and individuals who were pregnant or had a malignant tumor or mental disorder were excluded. The final sample size included 11,345 participants (5253 males and 6092 females). Participants in our study belonged to natural population who could be healthy people, diabetics, hypertensive, and participants suffering from cardiovascular diseases or other morbidities.

### Lifestyle factors

Information on covariates such as age, gender, and lifestyle, was collected during a single clinic visit by cardiologists and trained nurses who used a standard questionnaire to conduct a face-to-face interview. Prior to conducting the survey, all eligible investigators were invited to attend a training session which covered topics including the purpose of the study, how to administer the questionnaire, the standard method of measurement, the importance of standardization, and study procedures. After completing the training session, each potential investigator was required to obtain a perfect score on a training test if they were to participate in the study. Additionally, the investigators received further instructions and support during periods of data collection. Each study participant’s race was classified as either Han or other (which included ethnic minorities such as Mongol and Manchu). Family income was classified as ≤ 5000, 5000–20,000, and > 20,000 CNY/year. Educational level was categorized as low (no schooling, incomplete primary education, and primary education), middle (3 or 4 years of secondary education), and high (college and university education). All study participants were asked whether or not they were currently smokers or drinkers.

### Blood pressure measurements

Based on the recommended American Heart Association protocol, blood pressure was measured three times at 2-min intervals after at least 5 min of rest using a standardized automatic electronic sphygmomanometer (HEM-907; Omron; Kyoto, Japan). The participants were advised to avoid caffeinated beverages and exercise for at least 30 min before the measurement. During the measurement, each participant was seated with their tested arm supported at the level of the heart. The mean of three blood pressure measurements was calculated and used in all analyses.

### Anthropometric measurements

Weight and height were measured to the nearest 0.1 kg and 0.1 cm, respectively, with the participant in light weight clothing and without shoes. WC was measured at the umbilicus using a non-elastic tape (to the nearest 0.1 cm), and with the participant in a standing posture at the end of a normal expiration. BMI was calculated as the individual’s weight in kilograms divided by the square of the height in meters. WHtR was calculated by dividing waist circumference by height.

ABSI was calculated using the following formula [18]: $\mathrm{ABSI} = \kern0.5em \frac{\mathrm{WC}}{\ {\mathrm{BMI}}^{2/3}{\mathrm{Height}}^{1/2}}$

BRI: was calculated using the formula [19]: $\mathrm{B}\mathrm{R}\mathrm{I} = 364.2-365.5\times \kern0.5em \sqrt{1-\left(\frac{{\left(\mathrm{W}\mathrm{C}/\left(2\uppi \right)\right)}^2}{{\left(0.5\mathrm{height}\right)}^2}\right)}$_._

### Serum analysis

A fasting blood sample was collected from each participant in the morning after at least 12 h of fasting. Blood samples were obtained from an antecubital vein and collected in Vacutainer® tubes containing EDTA. Values for fasting plasma glucose (FPG), total cholesterol (TC), low-density lipoprotein cholesterol (LDL-C), high-density lipoprotein cholesterol (HDL-C), triglyceride (TG), and other routine blood biochemical indexes were obtained using an autoanalyzer. All laboratory equipment was calibrated, and blinded duplicate samples were used.

### Definition of DM

DM was diagnosed according to WHO criteria [24]: FPG ≥ 7 mmol/L (126 mg/dL) and/or being treated for diabetes.

### Statistical analyses

The general characteristics of diabetics and nondiabetics were compared using the non-parametric test for continuous variables and *χ*2 for categorical variables. The relationship between DM and ABSI, BMI, BRI, WC, and WHtR was examined using Spearman rank test. We used the area under the receiver-operating characteristic curve (AUC) and 95 % confidence intervals (CIs) to assess the discriminatory power of each anthropometric measure to assess the risk for DM. Quartiles of BMI, BRI and WHtR were created and the prevalence of DM was calculated in each quartile [23]. Since ABSI was strongly correlated with age and sex [18], ABSI was stratified for four age groups (age 35–44, age 45–54, age 55–64 and age **≥** 65), after which ABSI quartiles were determined within each age group for males and females separately. To calculate the prevalence of DM per ABSI quartile, subjects within the same ABSI quartile were merged [23]. For WC the quartiles were stratified by sex and the prevalence of DM in each quartile was calculated [23]. The odds ratios (ORs) and their 95 % CIs for the presence of DM were compared using the highest to the lowest quartile of each anthropometric measurement index, and were estimated by logistic regression analysis with adjustments made for age, race, family income, education, smoking, and alcohol status. All the statistical analyses were performed using SPSS Statistics for Windows, Version 17.0. Chicago, IL: SPSS, and *P*-values < 0.05 were considered statistically significant.

## Results

A total of 11,345 subjects (5253 males and 6092 females) aged ≥ 35 years participated in the study. There were 1182 participants suffering from diabetes (accounting for 10.4 % of our participants). Additionally, there were 1088 participants meeting FPG ≥ 7 mmol/L, and 548 participants were informed diagnosed DM. 451 participants were treated for DM, of which 94 participants were treated to goal. So, the prevalence of DM was 10.4 %, and the awareness, treatment and control rates were 46.4 %, 38.2 % and 8.0 % respectively among the rural populations in northeast China. These population prevalences are given and analyzed in more detail in another paper by our research group [25].

Table 1 shows clinical and demographic characteristics of the study population (diabetics vs. nondiabetics). The average age was 53.8 ± 10.6, and diabetics were significantly older (57.5 ± 9.7 years). Over all, the educational levels and family incomes in the rural areas were low. Additionally, the vast majority of participants were of Han nationality. All of the five anthropometric indices were larger in diabetics than those in nondiabetics. Furthermore, values for systolic blood pressure, diastolic blood pressure, uric acid, total cholesterol, triacylglycerol, low-density lipoprotein cholesterol and FPG were significantly higher in diabetics compared to those in nondiabetics, while the values for high-density lipoprotein cholesterol showed the opposite trend. Additionally, diabetics were less likely to be smokers or drinkers maybe because they quitted smoking or drinking after diagnosed with DM.

**Table 1:** Baseline characteristics of the study population

Variables	Totel (*N* = 11,345)	Nondiabetics (*n* = 10,163)	Diabetics (*n* = 1182)	*P*-value
Age (years)	53.8 ± 10.6	53.4 ± 10.6	57.5 ± 9.7	<0.001
Man (%)	5253 (46.3)	4731 (46.6)	522 (44.2)	0.12
Education				<0.001
Low	5760 (50.8)	4960 (48.8)	692 (58.5)	
Middle	4539 (38.4)	4231 (41.6)	392 (33.2)	
High	1046 (9.2)	972 (9.6)	98 (8.3)	
Family income (dollar/year)				0.06
<5000	1607 (14.2)	1238 (12.2)	166 (14.0)	
5000–20,000	6060 (53.4)	5547 (54.6)	646 (54.7)	
>20,000	3678 (32.4)	3378 (33.2)	370 (31.3)	
Smokers (%)	4007 (35.3)	3647 (35.9)	360 (30.5)	<0.001
Drinkers (%)	2565 (22.6)	2311 (22.7)	254 (21.5)	0.32
Han (%)	10,759 (94.8)	9640 (94.9)	1119 (94.7)	0.79
Others^a^ (%)	586 (5.2)	523 (5.1)	63 (5.3)	
Anthropometric measures				
Height (m)	1.61 ± 0.08	1.61 ± 0.08	1.60 ± 0.08	<0.001
BMI (kg/m^2^)	24.8 ± 3.7	24.6 ± 3.6	26.2 ± 3.7	<0.001
WC (cm)	82.4 ± 9.8	81.9 ± 9.7	87.1 ± 9.5	<0.001
ABSI (m^11/6^ kg^−2/3^)	0.0767 ± 0.0052	0.0765 ± 0.0052	0.0783 ± 0.0048	<0.001
BRI	3.70 ± 1.22	3.49 ± 1.11	3.87 ± 1.29	<0.001
WHtR	0.51 ± 0.06	0.51 ± 0.06	0.55 ± 0.06	<0.001
Measurement indicators				
Uric acid (mg/dL)	291.9 ± 84.8	290.9 ± 84.0	300.1 ± 91.4	<0.001
SBP (mmHg)	141.7 ± 23.4	140.3 ± 23.0	153.2 ± 24.0	<0.001
DBP (mmHg)	82.0 ± 11.8	81.6 ± 11.6	85.5 ± 12.5	<0.001
LDL-c (mmol/L)	2.9 ± 0.8	2.9 ± 0.8	3.2 ± 0.9	<0.001
HDL-c (mmol/L)	1.4 ± 0.4	1.4 ± 0.4	1.3 ± 0.3	<0.001
TG (mmol/L)	1.6 ± 1.5	1.5 ± 1.3	2.5 ± 2.6	<0.001
TC (mmol/L)	5.2 ± 1.1	5.2 ± 1.1	5.6 ± 1.3	<0.001
FPG (mmol/L)	5.9 ± 1.6	5.5 ± 0.6	9.3 ± 3.2	<0.001

*Abbreviations*: *ABSI* a body shape index, *BMI* body mass index, *BRI* body roundness index, *CNY* China Yuan (1CNY = 0.161 USD), *FPG* fasting plasma glucose, *HDL-C* high-density lipoprotein cholesterol, *LDL-C* low-density lipoprotein cholesterol, *DBP* diastolic blood pressure, *SBP* systolic blood pressure, *SD* standard deviation, *TC* total cholesterol, *TG* triacylglycerol, *WC* waist circumference, *WHtR* waist-to-height ratio

^a^Including some ethnic minorities in China, such as Mongol and Manchu

Table 2 shows the results of Spearman rank test of anthropometric measures (ABSI, BMI, BRI, WC, and WHtR) and DM. WHtR and BRI showed the highest Spearman correlation coefficient for DM (*r* = 0.163 for men and *r* = 0.183 for women, both *p* < 0.001) whereas ABSI showed the lowest (*r* = 0.110 for men and *r* = 0.121 for women, both *p* < 0.001) in both sexes. The point biserial correlation of DM with anthropometric variables was also calculated, as possibly more appropriate than Spearman rank correlation given the dichotomous nature of DM presence/absence, and yielded very similar coefficients and p values (not shown).

**Table 2:** Spearman rank test of anthropometric measures (ABSI, BMI, BRI, WC, and WHtR)^a^ and DM

Men (*n* = 5253)	ABSI	BMI	BRI	WC	WHtR
Diabetes	0.110	0.130	0.163	0.156	0.163
*P* value	<0.001	0.009	<0.001	<0.001	<0.001
Women (*n* = 6092)	ABSI	BMI	BRI	WC	WHtR
Diabetes	0.121	0.134	0.183	0.176	0.183
*P* value	<0.001	0.003	<0.001	<0.001	<0.001

*Abbreviations*: *ABSI* a body shape index, *BMI* body mass index, *BRI* body roundness index, *DM* diabetes mellitus, *WC* waist circumference, *WHtR* waist-to-height ratio

^a^Independent variable for all models

Table 3 shows the prevalence of DM in quartiles of ABSI, BRI, BMI, WC and WHtR.

**Table 3:** Prevalence of DM in quartiles of ABSI, BMI, BRI, WC and WHtR

Quartile (Men)	ABSI	BMI	BRI	WC	WHtR
1 (*n* of DM [%])	86 (6.5)	71 (5.5)	63 (4.5)	71 (5.1)	73 (4.7)
2 (*n* of DM [%])	100 (7.6)	107 (7.5)	134 (7.9)	79 (7.4)	108 (7.8)
3 (*n* of DM [%])	144 (11.0)	154 (11.9)	116 (12.9)	152 (10.3)	151 (12.6)
4 (*n* of DM [%])	192 (14.7)	190 (15.1)	209 (16.8)	220 (16.8)	190 (17.3)
Quartile (Women)	ABSI	BMI	BRI	WC	WHtR
1 (*n* of DM [%])	121 (7.9)	98 (6.5)	39 (3.5)	82 (4.9)	47 (3.7)
2 (*n* of DM [%])	156 (10.1)	124 (8.2)	125 (7.3)	109 (7.7)	96 (7.0)
3 (*n* of DM [%])	186 (12.4)	154 (10.4)	132 (11.4)	185 (12.8)	172 (11.2)
4 (*n* of DM [%])	197 (12.9)	284 (17.7)	364 (17.3)	284 (18.2)	345 (17.9)

Data presented as number (proportion)

*Abbreviations*: *ABSI* a body shape index, *BMI* body mass index, *BRI* body roundness index, *DM* diabetes mellitus, *WC* waist circumference, *WHtR* waist-to-height ratio

The prevalence of DM increased per quartile for all four anthropometric indices (1st quartile vs. 4th quartile) in both sexes: ABSI 6.5 % vs. 14.7 % for men, ABSI 7.9 % vs. 12.9 % for women; BMI 5.5 % vs. 15.1 % for men, BMI 6.5 % vs. 17.7 % for women; BRI 4.5 % vs. 16.8 % for men, BRI 3.5 % vs. 17.3 % for women; WC 5.1 % vs. 16.8 % for men, WC 4.9 % vs. 18.2 % for women; WHtR 4.7 % vs. 17.3 % for men, WHtR 3.7 % vs. 17.9 % for women (all *P* < 0.05).

Table 4 shows the multivariate-adjusted odds ratios (95 % CIs) of the presence of DM for each anthropometric index. Over all, the ORs of DM increased with increasing quartiles for all the five anthropometric indices, after adjustment for age, race, family income, education, smoking, and alcohol status. Considering the ORs of the presence of DM for the highest quartile vs. the lowest quartile of each anthropometric measure, the WHtR was the best predictor of DM (OR: 2.40, 95 % CI: 1.42–3.39 in men; OR: 2.67, 95 % CI: 1.60–3.74 in women, both *P* < 0.001), and the ABSI was the poorest predictor of DM (OR: 1.51, 95 % CI: 1.05–1.97 in men; OR: 1.55, 95 % CI: 1.07–2.04 in women, both *P* < 0.05). Compared to the ABSI, the BRI was a better predictor for DM in both sexes (OR: 1.79, 95 % CI: 1.31–2.27 in men; OR: 1.91, 95 % CI: 1.32–2.50 in women, both *P* < 0.01).

**Table 4:** Odds ratio (95 % CI) of the presence of DM for each anthropometric measure^a^

Quartile (Men)	ABSI	BMI	BRI	WC	WHtR
1 (reference)	1	1	1	1	1
2	0.95 (0.70, 1.2)	1.15 (0.81, 1.49)	1.30 (0.92, 1.68)	0.97 (0.60, 1.33)	1.54 (0.93, 2.14)
3	1.45 (0.99, 1.90)	1.31 (0.82, 1.80)	1.29 (0.81, 1.77)	1.24 (0.70, 1.77)	1.95 (1.05, 2.85)^**^
4	1.51 (1.05, 1.97)^*^	1.57 (1.06, 2.08)^*^	1.79 (1.31, 2.27)^**^	1.79 (1.31, 2.27)^***^	2.40 (1.42, 3.39)^***^
Quartile (Women)	ABSI	BMI	BRI	WC	WHtR
1 (reference)	1	1	1	1	1
2	1.27 (0.98, 1.56)	0.93 (0.69, 1.8)	1.59 (0.79,2.38)	1.23 (0.85, 1.60)	1.33 (0.79, 1.88)
3	1.45 (0.97, 1.94)	1.49 (1.00, 1.98)	1.84 (1.01,2.68)^*^	1.29 (0.91, 1.67)	1.58 (0.84, 2.32)
4	1.55 (1.07, 2.04)^*^	1.57 (1.07, 2.06)^**^	1.91 (1.32, 2.50)^**^	1.90 (1.32, 2.49)^***^	2.67 (1.60, 3.74)^***^

The between cut points are 0.0736, 0.0764, and 0.0794 for ABSI (men); 0.0732, 0.0764, and 0.0798 for ABSI (women); 22.2, 24.6, and 27.0 for BMI; 2.81, 3.55, and 4.34 for BRI; 0.77, 0.83, and 0.90 for WC (men); 0.75, 0.81, and 0.87 for WC (women); 0.47, 0.51, and 0.55 for WHtR

*Abbreviations*: *ABSI* a body shape index, *BMI* body mass index, *BRI* body roundness index, *DM* diabetes mellitus, *WC* waist circumference, *WHtR* waist-to-height ratio

^*^ *p* < 0.05, **p < 0.01, ***p < 0.001

^a^Adjusted for age, race, family income, education, smoking, and alcohol status

**Fig. 1:**
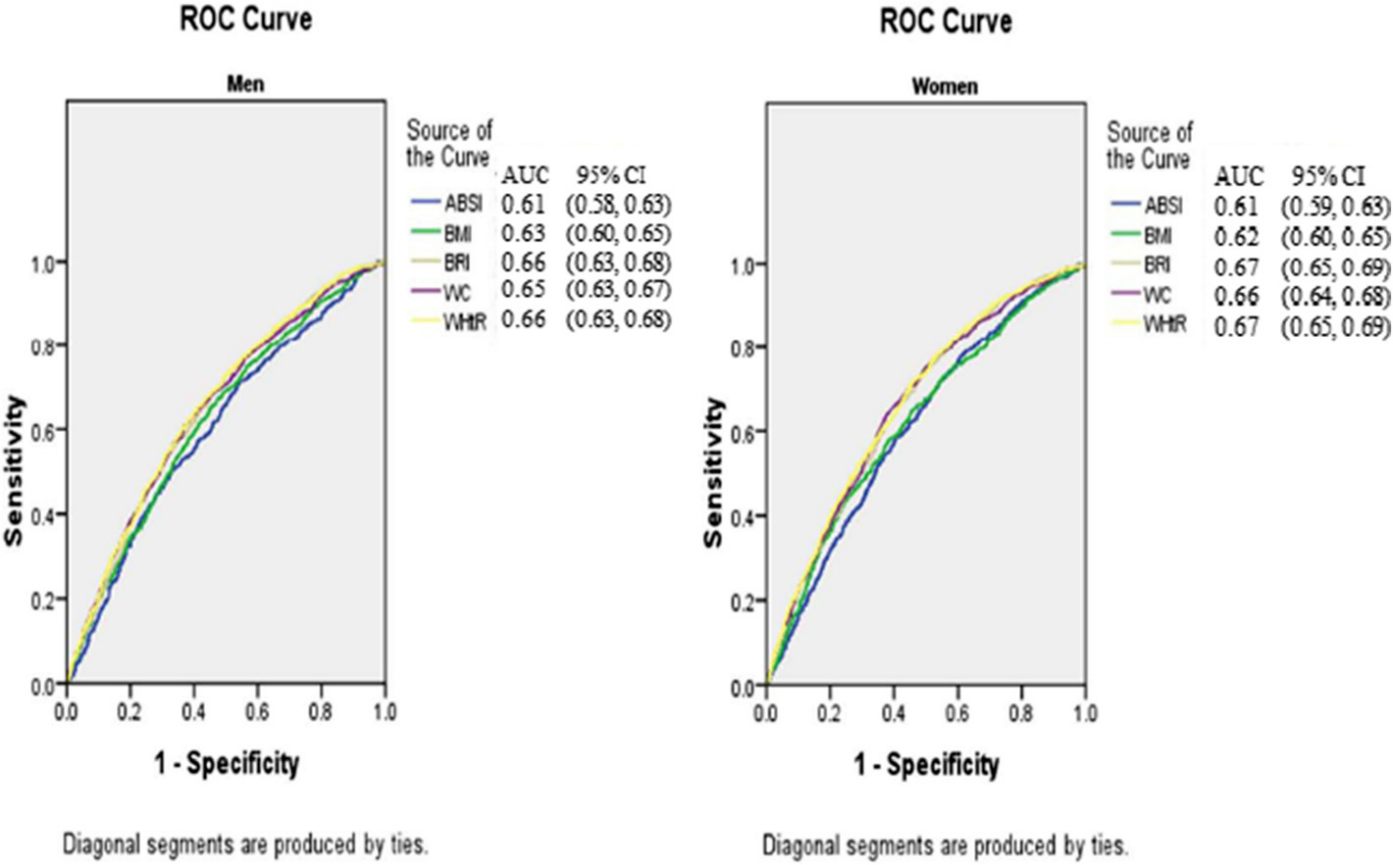
The discriminatory power of ABSI, BRI, BMI, WC and WHtR between subjects with or without DM. Area under the Receiver Operating Characteristic curve of ABSI, BRI, BMI, WC and WHtR to identify subjects with DM according to sex. Abbreviations: ABSI, A Body Shape Index; BMI, body mass index; BRI, body roundness index; DM, diabetes mellitus; WC, waist circumference; WHtR, waist-to-height ratio

Table 5 and Fig. 1 show the AUCs (and 95 % CIs) of anthropometric measures in the prediction of DM. Overall, BRI, WHtR, and WC were highly and almost equally predictive for DM. ABSI had the lowest AUCs for DM in both sex categories (AUC: 0.61, 95 % CI: 0.58–0.63 for men; AUC: 0.61, 95 % CI: 0.59–0.63 for women), while the BRI and WHtR showed the highest AUC values for DM in both men (AUC: 0.66, 95 % CI: 0.63–0.68) and women (AUC: 0.67, 95 % CI: 0.65–0.69).

**Table 5:** The area under the curve of each anthropometric measure for the presence of DM in both genders

Index	Men (*n* = 5253)	Women (*n* = 6092)
ABSI	0.61 (0.58, 0.63)	0.61 (0.59, 0.63)
BMI	0.63 (0.60, 0.65)	0.62 (0.60, 0.65)
BRI	0.66 (0.63, 0.68)	0.67 (0.65, 0.69)
WC	0.65 (0.63, 0.67)	0.66 (0.64, 0.68)
WHtR	0.66 (0.63, 0.68)	0.67 (0.65, 0.69)

*Abbreviations*: *ABSI* a body shape index, *BMI* body mass index, *BRI* body roundness index, *DM* diabetes mellitus, *WC* waist circumference, *WHtR* waist-to-height ratio

## Discussion

In this cross-sectional study, we compared two new anthropometric indices (ABSI and BRI) with other measurement indices for their usefulness in screening rural populations in northeast China who were at a risk for DM. Our results showed that all the five anthropometric indices could identify DM after adjustments for age, race, family income, education, smoking, and alcohol status. However, neither the ABSI nor BRI was superior to the BMI, WC or WHtR for determining the presence of DM. While our study indicated that the new anthropometric index ABSI was not suitable for identifying DM, another anthropometric index (BRI) showed capabilities similar to those of WHtR and WC.

### A body shape index

Krakauer et al. demonstrated that the ABSI was independent of height, weight, and BMI, and also found that the ABSI was more predictive for premature mortality than either BMI or WC [18]. As indicated in another study by Krakauer, ABSI was a readily computed dynamic gender and age sensitive indicator of mortality risk across BMI categories and had potential uses for making clinical decisions and for correlation with lifestyle and with other risk factors and health outcomes [26]. However, Maessen MF et al. found that ABSI was not a suitable measurement to identify either CVD or CVD risk factors [23]. He et al. in one perspective study conducted in China found that ABSI, BMI, and WC had similar predictive abilities for new onset DM after analyzing 15 years of follow-up data, suggesting that the ABSI was not better than WC or BMI for predicting DM [20]. Similarly, Fujita M et al. in one retrospective cohort study found that compared with BMI or WC, ABSI was not a better predictor of diabetes, hypertension, and dyslipidemia in Japanese adults [27]. Consistent with the studies from Western and Asian countries [20, 22, 23, 27], our study also found that after adjusting for age, race, family income, education, smoking, and alcohol status, ABSI showed the weakest predictive power for DM compared to other anthropometric indices. The ABSI was initially developed to predict mortality hazard in a follow-up study, and we applied it to predict DM in a cross-sectional study, which may be one reason why the ABSI failed to show superior predictive power. Furthermore, as suggested by other studies [20, 23, 27], we believe the main reason for these discrepant results was ethnic differences and (or) subject characteristics. During the face-to-face interviews conducted in our study, we found that people residing in rural areas had retained their traditional lifestyles. Both men and women of all ages reported engaging in agricultural activities which ranged from sowing in the spring to harvesting in autumn, and gathering firewood in the winter. The intensity of these activities was great enough that individuals tended to have better physical health compared to other less active individuals who had the same BMI. As suggested that ABSI could discern between fat and lean mass [18], the values of ABSI in our study were smaller than those in Krakauer’s study (0.0767 ± 0.0052 vs. 0.0816 ± 0.0053). In our study, some participants in the total group classified as obese by their BMI, could be classified as normal if evaluated by their ABSI. In other words, there were 875 obese people (345 in men and 530 in women, respectively) evaluated by their BMI, accounting for 7.7 % of the total people. This obesity prevalence would be relatively lower if the criterion was based on ABSI. That might be one reason why ABSI showed the lowest reliability for predicting DM, which is closely associated with obesity [2, 3]. Furthermore, as reported in Krakauer’s study, ABSI was strongly correlated with age and sex [18], which was further confirmed by subsequent studies. Xu et al. [28] suggested that the appropriate scaling exponents for standardizing waist circumference for BMI and height in Chinese adolescents were 0.45 and 0.55, respectively. Another study conducted in middle-age and older Indonesian population found that the regression coefficients for men were roughly similar to those reported in Krakauer’s study, but regression coefficients for women were different [29]; suggesting that the appropriate scaling exponents should be modified for gender. Finally, the equations used to calculate the ABSI for men and women were modified as follows: $\mathrm{ABSI} = \kern0.5em \frac{\mathrm{WC}}{{\mathrm{BMI}}^{2/3}{\mathrm{Height}}^{1/2}}$ for men and $\mathrm{ABSI} = \kern0.5em \frac{\mathrm{WC}}{{\mathrm{BMI}}^{3/5}{\mathrm{Height}}^{1/5}}$ for women.

Although the precise reasons for the discrepant results were unascertained, ABSI was defined to be independent of BMI and thus could be an important complement to it when identifying subjects at risk of some diseases.

### Body roundness index

Thomas et al. [19] first developed the BRI to predict body fat, the percentage of visceral adipose tissue, and establish an initial impression of an individual’s physical health. Up to now, only one study has used the BRI to predict disease, and showed that the BRI was capable of identifying both CVD and CVD risk factors. Additionally, the adjusted OR of the BRI was higher than those of the BMI and WC, although the differences were not statistically significant [23]. However, inconsistent with the above study [23], our results demonstrated that while BRI was able to identify DM, it did not show superior predictive power when compared to WC or the WHtR. Furthermore, the values for height, WC, BMI, ABSI and BRI were all lower in our study compared to those in the previous study [23]. Thomas et al. [19] demonstrated that values for BRI can reflect both visceral adipose tissue (VAT) and % body fat, and thus the smaller values for BRI in our study may indicate that the participants had lower amounts of subcutaneous adipose tissue. This is consistent with the above-stated conclusion that smaller values for ABSI indicated a higher deposition of muscular tissue and a lower deposition of visceral adipose tissue in a rural population. Furthermore, as Maessen MF et al. [23] demonstrated in their study that the Spearman rank test revealed a perfect nonlinear relation between BRI and WHtR (*r* = 1; *P* = 0.00). Our data analysis showed an exactly coincident results (*r* = 1; *P* = 0.00) using the spearman rank test, which indicated that both body indices are closely related. From its definition, BRI is a one-to-one nonlinear transformation of the WHtR. Therefore, ranks for BRI in the population are the same as for WHtR, and the Spearman rank correlation between BRI and WHtR is exactly 1. The reason for the different numbers and odds ratios of each quartile in Table 3 for BRI as compared with WHtR appears to be simply that we rounded the inter-quartile thresholds to two decimal places before applying them to each index. Furthermore, in spite of some shortcomings as demonstrated by Thomas et al. [19], the advantage of the BRI over the WHtR is that it also can be used to estimate the amount of body fat percentage and gives therefore a better impression of physical health status. In conclusion, the BRI showed a superior predictive capacity compared to the ABSI and BMI, and demonstrated potential for improving the detection and evaluation of DM.

### Limitations

This study has some limitations that should be mentioned. First, the ABSI was initially developed to predict mortality hazard in a follow-up study, and we applied it to predict DM in a cross-sectional study, which may be the main reason why the ABSI failed to show superior predictive power. The longitudinal relationship between the two new anthropometric indices and DM should be examined in future studies. Second, our study was conducted with rural populations residing in northeast China, and the unique lifestyle of those populations may have influenced the body shape and metabolic indices of the participants. As previously mentioned, the two new anthropometric indices were first developed in Western countries (both in America), and should be modified as suggested by other studies [27, 28] to make them suitable for use with Chinese populations having different characteristics.

## Conclusions

Our present study found that neither the ABSI nor BRI was superior to BMI, WC, or the WHtR for predicting the presence of DM. The ABSI showed the weakest predictive power, while the BRI might be used as an alternative obesity measure for assessing DM. We hope that more studies are conducted which examine the advantages and disadvantages of these new anthropometric indices.
